# Living With *Ma’i Suka*: Individual, Familial, Cultural, and Environmental Stress Among Patients With Type 2 Diabetes Mellitus and Their Caregivers in American Samoa

**Published:** 2008-06-15

**Authors:** Emily Elstad, Corabelle Tusiofo, Rochelle K Rosen, Stephen T McGarvey

**Affiliations:** New England Research Institutes. At the time of this study, Ms Elstad was affiliated with Brown University, Providence, Rhode Island; Tafuna, American Samoa; The Miriam Hospital, Providence, Rhode Island; International Health Institute, Brown University, Providence, Rhode Island

## Abstract

**Introduction:**

The U.S. territory of American Samoa has a disproportionate number of people with type 2 diabetes mellitus compared with neighboring Samoa and the U.S. mainland. The purpose of this research was to study perceptions of diabetes among people with type 2 diabetes in American Samoa in order to design culturally appropriate interventions to prevent and manage diabetes effectively.

**Methods:**

Seven focus groups were held with 64 participants at a primary health care facility and a nearby workplace in American Samoa. These focus groups were conducted in the Samoan language and explored perceptions of diabetes, including its meaning, etiology, and the illness experience. Participants were people with diabetes at the health care facility and their family caregivers.

**Results:**

Our systematic analysis of the translated transcripts showed that American Samoans with type 2 diabetes experienced individual, familial, cultural, and environmental stress. They also associated environmental and familial stressors with the worsening of symptoms and increases in blood glucose levels. Although participants believed that stress within the family worsened diabetes symptoms, family members figured prominently as primary caregivers.

**Conclusion:**

Interventions aimed at improving diabetes management in American Samoa should emphasize family involvement coupled with education and methods to reduce caregiver burden, given the chronic, lifelong nature of diabetes.

## Introduction

Epidemic rates of type 2 diabetes mellitus are being reported for Pacific Islanders, and in the U.S. territory of American Samoa, a small island in the Western Pacific Region, the high prevalence of diabetes has increased. Among adults aged 25 to 55 years in 1990, 12.9% of men and 8.1% of women had fasting serum glucose levels of ≥126 mg/dL or were taking medications for type 2 diabetes; in 2002, 17.2% of men and 16.7% of women had type 2 diabetes, using the same criteria (1-3). A 2004 World Health Organization survey found that 52.3% of men and 42.4% of women aged 25 to 64 years in American Samoa had type 2 diabetes (4). The report was based on respondents' self-reports that they had received a diagnosis of diabetes from a health care provider, took diabetes medications, or had a fasting glucose level of ≥110 mg/dL. Regardless of the exact criteria used, these data indicate that diabetes prevalence in American Samoa is much higher than the U.S. diabetes prevalence of 10.5% for men aged 20 or older and 8.8% for women in the same age bracket (5).

Economic development in American Samoa has increased since the 1950s, yet the country remains poor by U.S. standards. Although per capita income for American Samoa rose from $596 in 1969 to $3039 in 1989 and $5800 in 2005 (6), the 2000 U.S. Census estimated that 61% of the population lives below the federal poverty guidelines (7). These rapid economic changes, common in the Pacific region, have led to a nutrition and health transition in American Samoa (3,8).

The economic development of American Samoa may be aptly compared to that of nearby Samoa, where the economy is based on agriculture and tourism. In Samoa, lifestyles are more physically demanding, fewer people are employed in sedentary jobs, and the population relies less on imported energy-dense processed foods than in American Samoa (3). In Samoa, prevalence rates of type 2 diabetes in 2003 were less than half those found in American Samoa in 2002 using the same criteria (3). These data suggest that economic development has played a role in increasing the rate of type 2 diabetes in American Samoa.

The contemporary diet in American Samoa is characterized by traditional foods high in fat and starch (e.g., coconut cream, taro) combined with processed foods also high in starch and fat, such as rice, mutton flaps, corned beef (9), snack foods, canned soda (3), and fast foods from McDonald's, Kentucky Fried Chicken, or Pizza Hut. In addition, economic changes have reduced physical activity. Samoans are increasingly employed in jobs that require less physical activity than the traditional livelihoods of precontact Samoa, such as fishing and preparing coconut meat (10,11). These macroenvironmental changes have greatly exacerbated structural and individual risk factors for developing type 2 diabetes in this population (1,3).

Health care providers in American Samoa have increased their emphasis on diabetes education of patients. The Centers for Disease Control and Prevention (CDC) funds a small diabetes control program, but until recently, the country had no scientific interventions aimed at improving diabetes management. Although one recent qualitative study has examined American Samoan perceptions of cancer (12), our study is the first qualitative research to examine American Samoan perceptions of diabetes.

Our research indicates the need for culturally specific interventions aimed at addressing the clear disparity in diabetes burden experienced by American Samoans. Resources for planned interventions in American Samoa differ from those in the United States. The country also has fewer specialty medical services for diabetes, fewer and more expensive food choices for people with diabetes, and limited opportunities for physical activity (4).

## Methods

This study took place at the Tafuna Family Health Center (TFHC), a primary health care center on the island of Tutuila. TFHC serves a district where more than a third of the population of American Samoa resides.

Our qualitative descriptive methodology used focus group interviews to explore the experiences of American Samoans with diabetes and their caregivers. Specifically, focus group questions aimed to assess participants' perceptions of diabetes, including its meaning, etiology, management, and the illness experience. Kleinman's Explanatory Model (13) suggests that health care providers can better communicate with patients by exploring the patients' understanding of an illness. This model informed the focus group questions, which were further developed by the research team with input on cultural appropriateness from the Samoan author (CT). Caregivers were not asked specific questions but were encouraged to contribute to the discussion. The focus group discussion agenda is provided as an appendix to this report (Appendix).

The data collection team consisted of one Samoan public health nurse experienced in diabetes management and trained as a focus group facilitator for this research (CT) and one of the authors (EE). Participants completed a consent form, and ethical clearance was granted by the Brown University Institutional Review Board (IRB) and the American Samoa Department of Health IRB for the study protocol.

From June through August 2005, we conducted 6 focus groups at TFHC and 1 at a nearby workplace. A convenience sample of people with diabetes (N = 110) drawn from TFHC's diabetes registry was invited to participate in the groups. The facilitator recruited participants for the focus groups by telephone. Each recruited individual was asked to bring 1 family member or friend who helped them manage their diabetes. Of the 110 people invited to join the focus groups, 64 attended. All unaccompanied participants were offered a proxy caregiver (a TFHC outreach worker); this approach was suggested by the focus group facilitator and second author as a way to make all unaccompanied participants feel equal and included. Three unaccompanied participants with diabetes in 3 focus groups elected to have a proxy caregiver. The size of focus groups ranged from 4 to 17 people (mean, 9 people) (Table 1). Focus groups lasted from 30 to 90 minutes (average duration, 61 minutes).

Participants were eligible if they met the following criteria: adult (aged 18 years or older); a male or female Samoan resident of American Samoa (self-defined); diagnosed with type 2 diabetes or a caregiver of someone with diabetes; fluent in either Samoan or English; and willing to participate in the focus group meeting. All but 2 participants spoke Samoan. Of the 64 individuals who participated in the study, 35 were women and 29 were men. Generally, the mix of men and women in each group was even. Of the 64 participants, 35 had diabetes. Eighteen of the 35 brought a caregiver with them, and 3 participants with diabetes elected to have a staff member participate as their proxy caregiver. In the case of the focus group held at a nearby workplace, 12 participants were invited to join the group because they had a family member with diabetes. Participants with diabetes were aged 30 to 84 years (mean age, 55 years); caregivers tended to be younger (aged 21–63 years; mean age, 41 years).

At the end of each session, participants were told they could stay to ask the facilitator — a licensed nurse practitioner — any questions or have their blood glucose level tested. Those who stayed were referred to additional resources for care. Participants in 3 of the 7 focus groups used this service. Because the group sessions were held around dinner time, participants were served a healthy and culturally appropriate meal of chicken, rice, vegetables, and bottled water.

Focus group sessions were audiotaped and transcribed from Samoan into English by the facilitator and note taker. Transcripts were first read by a team of 2 qualitative researchers to identify initial themes and develop codes. Transcripts were then read more carefully to refine the coding structure. Transcripts were coded for the selected themes using NVivo 2.0 software (QSR International, Doncaster, Australia). Both normative and outlying responses were considered germane and were coded.

Characteristics of participants with diabetes varied by focus group (Table 2). Fifteen (43%) were men, and 20 (57%) were women. Caregivers were predominantly female by a ratio of 4:1. Three caregivers from the first 2 focus groups also had diabetes. Seventeen people with diabetes in 5 focus groups attended without a caregiver because they either chose not to bring one or because of scheduling conflicts. One unaccompanied participant with diabetes in each of 3 focus groups opted to have an outreach worker participate as their proxy caregiver for a total of 3 proxy caregiver participants in the sample. Fourteen unaccompanied participants with diabetes opted not to have a proxy caregiver. Outreach workers shared only their personal experiences and were asked not to share their knowledge of diabetes etiology during the discussion. Because family caregivers did not exact notable influence over people with diabetes (e.g., facilitate participation in discussion), the absence of family caregivers and the presence of TFHC proxy caregivers did not noticeably change the dynamic of the focus groups.

## Results

### Stress and diabetes in American Samoa

Although stress was not a domain of primary interest written into the research agenda (see Appendix), the issue of diabetes-related stress emerged strongly in response to the question, "What prevents you from managing your diabetes?" Our research found that Samoan participants experienced diabetes-related stress on a number of levels. We have placed participants with diabetes in a multitiered context as individuals, family members (immediate and extended), and Samoans living with cultural and environmental stress as part of the structure of a changing nation.

**Individual stress**

Many participants defined diabetes (*ma'i suka* in Samoan, literally, "sugar disease") as the sum of their symptoms. The predominant phenomenological vehicle for defining diabetes in our focus groups was through what Cassell (14) calls an "alien bodily sensation":

Diabetes . . . is when you're always sleepy and wanting to eat all the time. (man with diabetes, aged 66)

That's another meaning of diabetes — getting headaches, dizziness, and body aches. (man with diabetes, aged 42)

Diabetes is the sensation of tingling in my fingers when my glucose is too high. (woman with diabetes, aged 55)

Participants' experiences of diabetes-related stress as individuals were shaped by moments of "suffered illness." Kleinman (13) holds that naming the illness experience — a process he refers to as the "search for diagnosis" — is not uncommon among people who regularly experience pain and discomfort. Because life for a person with diabetes is regularly disrupted by pain and discomfort, it is not surprising that he or she should name that experience.

Similar findings were recorded among American Indian women with diabetes in a study by Taylor et al (15), and a recent study in American Samoa found that cancer was also defined through its signs and symptoms (12). Likewise, when asked what diabetes means, many respondents in our study defined their disease through their felt symptoms and the personal stress these symptoms caused:

My side was hurting so bad — especially after drinking soda — that I had to go to the doctor. The results came back with a very high glucose level. . . . That is my understanding of what diabetes is. It's when you feel bad because you don't eat the food you're supposed to eat. (woman with diabetes, aged 57)

The participant quoted above demonstrates that the experience of "feeling bad" — that is, the experience of pain and discomfort — itself constitutes the intrapersonal stress of living with diabetes. However, interpersonal stress figured most prominently in our focus groups.

**Familial stress**

Tamasese et al observe, "You cannot take a Samoan out of the collective context" (16), and indeed, in our research, the relationship between stress and diabetes was often familial in context. For the focus group participants, stress from family relationships was related to increased and aggravated symptoms of diabetes. Participants often attributed their worsened symptoms to a spouse or children:

Sometimes my glucose goes up to at least 200 [mg/dL] or more because of the old lady and my kids. . . .  Worries and problems within the family . . . contribute to the glucose going up. (man with diabetes, aged 66)

When the kids give you a headache, your glucose and your blood pressure will go up! . . . This is one of the things that worsens my disease [and makes it] hard to control my diabetes. (woman with diabetes, aged 72)

Other people with diabetes blamed their anger on the worsening of symptoms, although family members were cited as the cause of this anger:

I yell at my kids and am angry all the time. Then my husband and my kids turn around and tell me, "That's why your glucose level goes up — because you're mad!" (woman with diabetes, aged 57)

I know this is one of the reasons why blood pressure and glucose go up — because of anger. (woman with diabetes, aged 48)

Samoan traditional culture may factor into the perception of insufficient care and its relationship to worsened symptoms, because in Samoan culture, family and community are traditionally responsible for promoting a quiet, still environment around the home of a sick person in an effort to contribute to the healing process (17). According to MacPherson and MacPherson (18),

[In Samoa], a person is permitted certain privileges when they are unwell . . . [and] an illness may also be a consequence of the performance of another person. . . . This usually occurs where obligations connected with a role are not performed adequately.

Friction within the extended family was also a cause of stress among people with diabetes. One participant mentioned that the Samoan tradition of *fa'alavelave* — family functions such as weddings or funerals to which relatives are obliged to give money — were especially stressful for him given his health condition:

When my family comes over, I tell them I have to go to my doctor's appointment! It stresses me out that I have to give up money for that purpose when I could use it for my family and for myself. They ask me, do you have any money for a donation? And . . . at that point my blood pressure and my glucose goes up! It causes imbalance! (man with diabetes, aged 66)

Physical, mental, and spiritual balance is extremely important to the Samoan notion of self, and the sense of imbalance this participant feels may indicate that something is vastly amiss (16). Further research is required to explore this notion that diabetes is testing the endurance of the *fa'aSamoa* or "Samoan way."

**Cultural and environmental stress**

Our research also places the Samoan with diabetes in a larger context of acculturation and stress. The effects of cultural stress, accumulated over time because of colonialism and acculturation, have been found to be at play in indigenous experiences of diabetes (19). Increased exposure to regional and global economic markets during the past half-century has made more non-Samoan food items available in American Samoa. These include packaged, frozen, and canned foods that are high in fat and refined sugar. An influx of fast-food chains has further changed the Samoan diet (3,9,20). This new diet, combined with other lifestyle factors such as decreased exercise and increased cigarette smoking (3), contributes to American Samoans' increased risk of developing noncommunicable diseases. Furthermore, the immunosuppressive effects of stress resulting from the "lifestyle incongruity" found in cultures experiencing rapid cultural and economic change is pronounced among Samoan adults (21) and adolescents (22).

Many participants in this study characterized diabetes as a new disease to American Samoa:

I am not used to [diabetes] from the old days. . . . Nowadays we just eat and sit. There is no more going outside to pull weeds or sweep the trash outside the house. This is probably why this disease came. (woman with diabetes, aged 72)

I used to be an active person, but when I came here to American Samoa from Western Samoa, I stopped being active because I had to work [at the tuna cannery]. (man with diabetes, aged 64)

In the change from subsistence living to a cash economy, American Samoans have become less active (10,11,20). MacPherson and MacPherson (18) note that in traditional Samoan culture, the process of food production was arduous, involving farming and fishing, which made obesity and its associated illnesses uncommon. Today, many American Samoans sit at work and drive to their destinations. One participant with diabetes described a scenario that has been possible only in recent years:

I get in front of the cashier and there is Snickers. I put 2 Snickers in my pocket! I joke to myself by saying, 'This is what my disease likes.' If not, I go with a package of cookies in my car and a Diet Coke. (man with diabetes, aged 46)

Fast food has also changed the face of American Samoa. In 2000, the first McDonald's was opened in American Samoa. It is across the road from a Kentucky Fried Chicken and a Pizza Hut. Two participants describe how fast food has changed the island:

Back in the day, we had fresh food, but nowadays we are so lazy that, most of the time, we go eat out at McDonald's or KFC. . . . And these restaurants use a lot of fat and grease in their cooking. (man with diabetes, aged 46)

The food that the adults eat, the kids don't like. Our children sometimes don't eat together with our family. . . . But . . . it's a custom here in Samoa for the family to eat together! . . . [The children] always cry for hamburgers! I always attempt to not give them McDonald's though because it's not good. (woman with diabetes, aged 56)

### Help with diabetes management

Focus group participants were asked who helps them manage their diabetes and how. People with diabetes were then asked to describe what their caregiver does to help them. The people who mainly helped focus group participants were their spouses and children. A few participants mentioned that their extended family helped, too. Participants reported that their family members helped them by reminding them to exercise or eat healthily, helping them get to appointments, reminding them to take medication, massaging their feet, or fetching things they cannot get themselves:

The wheelchair doesn't move by itself, so [my son] always pushes me to the table to make sure I eat and makes sure I get my medication. [My son] is a big help, he helps me and cares for me. (man with diabetes, aged 70)

My husband helps me a lot. Whenever we argue about me not wanting to exercise [or] eat what he cooks for me, my husband gets so mad! (woman with diabetes, aged 57)

The following comments, made by caregivers, reflect the other side of the equation:

The best way to help my husband is to remind him when to take his medication because sometimes he forgets. (woman without diabetes, aged 38)

My mother was also a diabetic and I used to care for her when she was alive. I used to prepare meals. . . .  Now I'm doing it for myself. . . . I have also trained my husband to take care of my diabetes, so now he is caring for me. (woman with diabetes, aged 44)

## Discussion

Our study found that the stress of living with diabetes was individual, familial, cultural, and environmental. This report provides an important context for understanding the complex struggle of living with diabetes in a collectivist society such as American Samoa. Our research also lays the groundwork for the design of culturally appropriate interventions to help manage diabetes in American Samoa.

Several limitations of this study should be mentioned. First, some meaning may have been lost in translation of the transcripts from Samoan to English; time constraints precluded back-translation or verification beyond the perspective of a single translator. Second, although we made every effort to keep all focus groups comparable, 1 of the 7 focus groups was held not at TFHC but at a nearby workplace, which may have introduced exposure bias. In addition, this particular focus group was much larger than the others, having 17 attendees, and caregivers outnumbered participants with diabetes because anyone who cared for a relative with diabetes was invited to attend. The different dynamic of this focus group should be taken into account. Third, outreach workers participated in 3 of the focus groups as proxy caregivers for 3 unaccompanied participants with diabetes, and although the outreach workers participated as equals and shared only personal experiences, their participation may have influenced the candidness of participant responses. We did not, however, notice any difference in the tone of the 3 focus groups in which they participated.

Because of the small sample size (n = 64) and the nature of the convenience recruitment and sampling, focus group participants may not be representative of all adults with type 2 diabetes in American Samoa. We are not aware of any procedures that would produce noteworthy sampling bias, except that unspecified illness prevented some people from participating. Some people with diabetes may have decided not to attend a focus group because they did not have a caregiver to bring with them. However, 17 unaccompanied people with diabetes (or almost 30% of the sample) did attend the focus groups.

To our knowledge, this study is the first qualitative research on American Samoan perceptions of diabetes. The stress-related findings are noteworthy in that they emerged across all focus groups in response to a question about barriers to diabetes management — not specifically about stress. Stress was found to be a major barrier to disease management and was associated with the worsening of symptoms. These findings provide insight into the multiple levels at which American Samoans with diabetes experience stress. Familial and cultural or environmental stress were particularly pronounced.

Stress within the family was by far the most cited example of a factor that makes disease management difficult, although family members were overwhelmingly named the primary caregivers to participants with diabetes. The importance of family in the process of healing in traditional Samoan culture has been noted (17,18). Our findings suggest that interventions aimed at improving diabetes management in this population should emphasize family involvement and education and methods to reduce caregiver burden, given the chronic, lifelong nature of diabetes. Further research is needed to capture family members' perspectives as caregivers of someone with diabetes. Additionally, our research suggests that the concept of self-management, often used to instill in people the skills needed to manage their own illnesses, might need to be expanded in this culture to include the role of the family and caregiver. Respondents' comments demonstrate that skills for managing diabetes are shared within the family and passed across generations. It follows that preventive behaviors — not just management skills — can be learned and shared.

One of the outcomes of stress caused by cultural or environmental changes is a drastically increased risk of chronic diseases such as diabetes. Such stress was studied in American Samoa previously and was found to be significantly associated with various biological health measures (e.g., blood pressure, urinary catecholamines, immune function indicators of stress) in the context of modernization and acculturation (22-26). Our research found that Samoan participants associated stress with worsening of symptoms, a result that has been recorded among other populations (27-31). Stress management and reduction may, therefore, be a key area for intervention.

Finally, our research found that diabetes is recognized by Samoans as a disease new to the nation and was associated with their perceptions of cultural or national changes in diet and exercise. The results of this study suggest that structural interventions emphasizing improvements in diet and exercise habits may be well received in American Samoa, especially by the older generation.

Previous studies have reported perceptions, notably among Hispanic or Latino populations, that stress and anger worsen diabetes symptoms (27-31), and a study from Sweden found that non-Swedish men connected emotional stress to development of diabetes and decline in health (31). Further research is required to clarify the physiological relationship between stress and diabetes symptoms.

MacPherson and MacPherson offer an explanation for the causal link some participants made between family-related stress and the worsening of symptoms: the insufficient fulfillment of a role or obligation on the part of another person. As older adults in the community, people with diabetes may feel entitled to a communal effort, especially from their immediate family, to help them get well (17). Chronic illness may confound this tradition. Sustaining special care throughout the life of a family member with diabetes may prove a difficult task for family members to undertake, and such efforts will never achieve a cure, only improved management. More research is needed to better define the relationship between chronic disease and caregiver burden in American Samoa.

Our respondents describe the ways in which the American Samoan diet has changed in the past half-century, the disruption caused by the influx of fast-food restaurants, and the generational divide in food preferences. Many participants linked the changes to their illness. Notably, one of the focus group participants challenged the focus group facilitators to tell him what diabetes was. "Back in the day," he said, "we didn't have this disease, but now we have it. So . . . you should be telling us what [diabetes] means, not us telling you!" His comment suggests a collective Western responsibility, if not for the Samoans' diabetes burden itself, then for education about the etiology and causation of the disease.

## Figures and Tables

**Table 1:** Characteristics of Participants, by Focus Group, American Samoa, 2005

Focus Group	Participants					Age	

	Total	With Diabetes	Caregivers (Proxy Caregivers)	Men	Women	People With Diabetes	Caregivers

						Range, y (mean)	Range, y (mean)
1	9	5	4 (0)	5	4	35-70 (58)	26-63 (46)
2	9	5	4 (0)	1	8	32-84 (55)	34-57 (42)
3	8	5	2 (1)	4	4	42-66 (51)	38-56 (48)
4	9	5	3 (1)	3	6	44-65 (56)	29-46 (40)
5	4	2	1 (1)	1	3	56-61 (59)	40-42 (41)
6	17	5	12 (0)	14	3	40-64 (49)	21-58 (37)
7	8	5	3 (0)	1	7	30-61 (55)	24-44 (33)
**Total**	64	35^a^	29 (3)	29	35	30-84 (55)	21-63 (41)

a Total includes 3 caregivers with diabetes.

**Table 2:** Characteristics of People With Diabetes, by Focus Group, American Samoa, 2005

Focus Group	Total** Participants^a^**	With Diabetes			

		Total	Men	Women	Unaccompanied by Caregiver
1	9	7	4	3	0
2	9	6	1	5	1
3	8	5	3	2	2
4	9	5	2	3	1
5	4	2	1	1	2
6	17	5	3	2	5
7	8	5	1	4	6
**Total**	64	35	15	20	17

a Total includes 3 caregivers with diabetes.

## Appendix. Interview Guide for Focus Groups of American Samoans With Type 2 Diabetes Mellitus, 2005

1. Definition: What is diabetes?
2. Diabetes etiology
  - What causes diabetes?
  - Who is at risk for getting diabetes?
3. Patient response to diabetes diagnosis: Please tell the story of how you found out you had diabetes.
  - How did the doctor explain diabetes to you? What did s/he tell you about the disease? What did you think it would mean to have diabetes? (Probe)
  - How did you feel when you found out you had diabetes? (Probe)
4. Self-management: What can you do to avoid the symptoms of diabetes and to keep your diabetes from getting worse?
5. Barriers to self-management: What prevents you from managing your diabetes?
6. Family participation in diabetes management
  - Who helps you the most with your diabetes?
  - How does this person help you?
7. Management programs: What programs would you like to see in your community to help you manage your diabetes?
