# Erratum, Vol. 5: Issue 3

**Published:** 2008-09-15

Table 2 in the article "Living With *Ma'i Suka*: Individual, Familial, Cultural, and Environmental Stress Among Patients With Type 2 Diabetes Mellitus and Their Caregivers in American Samoa," which appeared in the July 2008 issue, was amended to include a footnote that was missing in the original publication. The footnote indicates that the column headed "Total Participants" includes 3 caregivers with diabetes. The changes were made June 25, 2008, and appear online at www.cdc.gov/pcd/issues/2008/jul/07_0101.htm.

